# Supplementary figures and images for: Understanding the progress of COVID-19 transmission in a rural district: a social network approach
Source: PeerJ. 2024 Nov 28;12:e18571. doi: 10.7717/peerj.18571 (PMC11608564; doi:10.7717/peerj.18571)

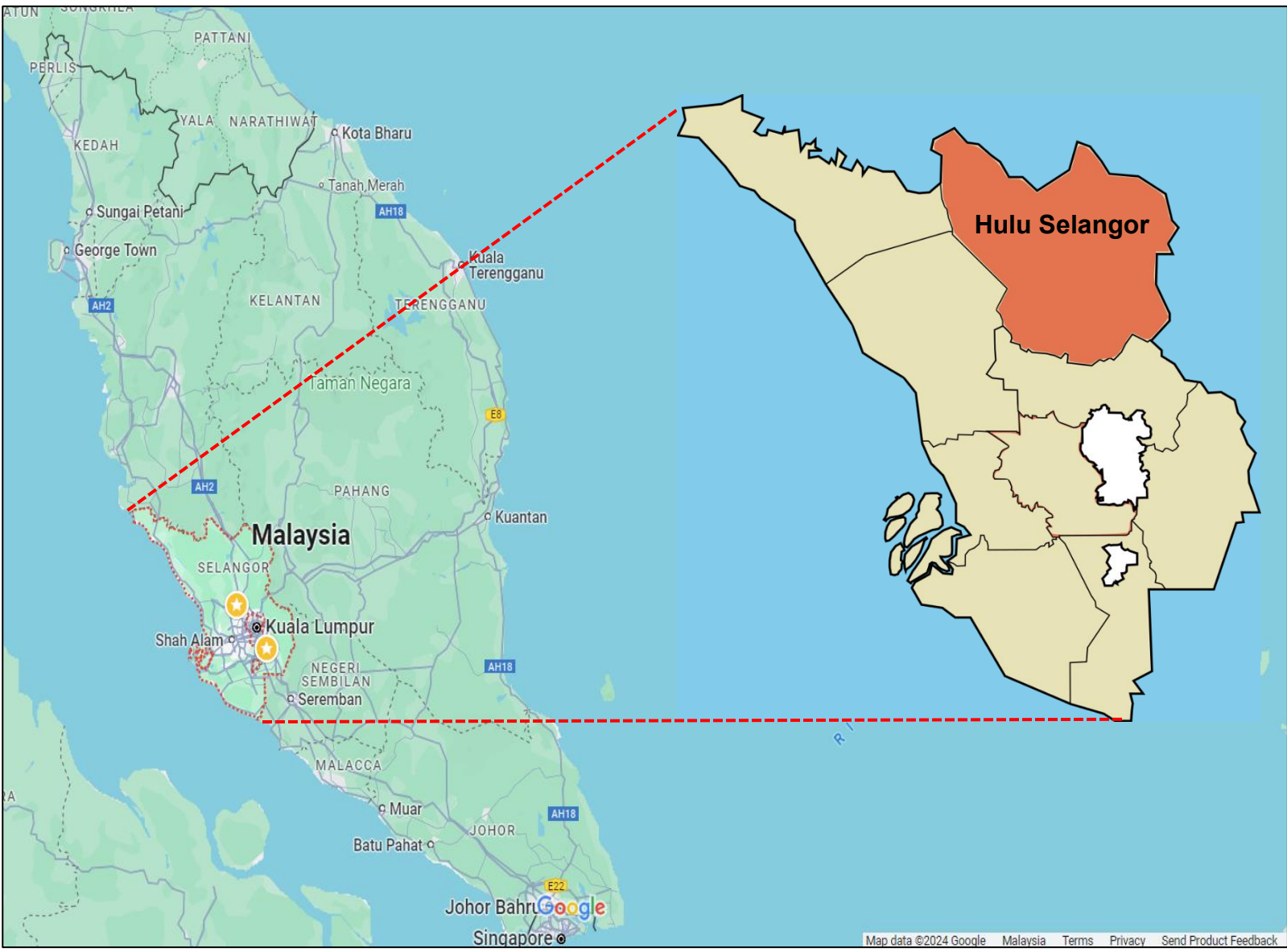

Supplement: Supplemental Information 1 — Location of the study area: Hulu Selangor district (colored area on the right-sided map) and Selangor state (left-sided map). Map data © 2024 (left map). Image credit: Bukhrin at https://commons.wikimedia.org/wiki/File:Daerah_Hulu_Selangor_Highlighted_in_the_State_of_Selangor,_Malaysia.svg (right map) [file peerj-12-18571-s001.pdf]

i) All positive case

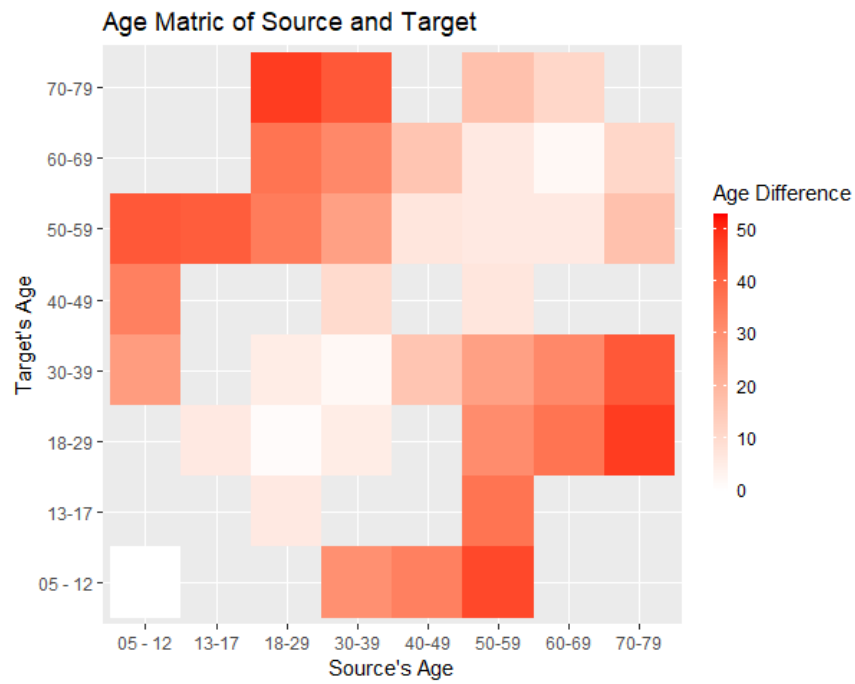

ii) Positive male cases

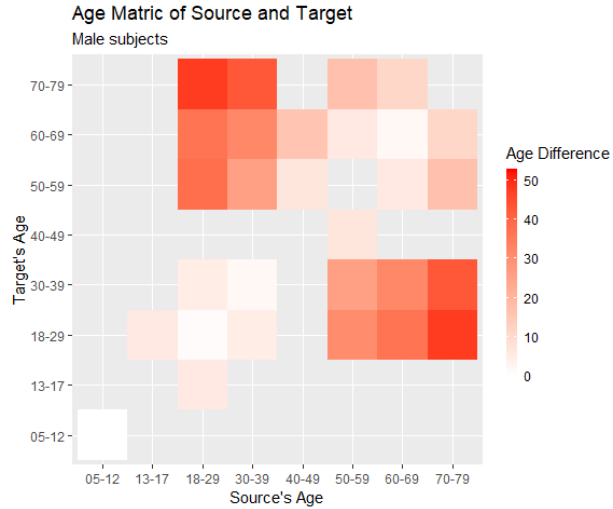

iii) Positive female cases

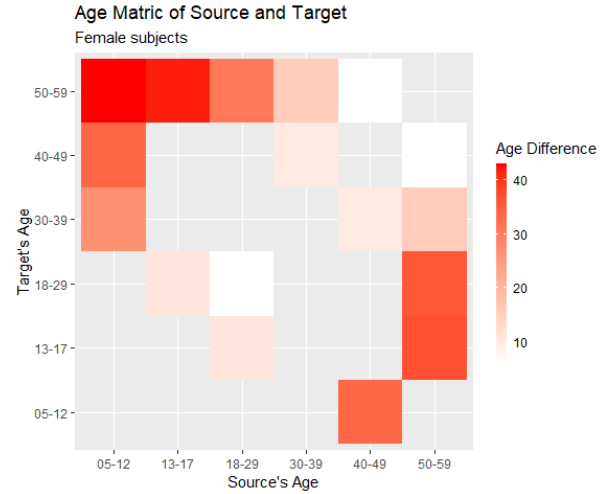

Supplement: Supplemental Information 5 [file peerj-12-18571-s005.pdf]
