# Supplementary material for: Understanding the progress of COVID-19 transmission in a rural district: a social network approach
Source: PeerJ. 2024 Nov 28;12:e18571. doi: 10.7717/peerj.18571 (PMC11608564; doi:10.7717/peerj.18571)
Supplement: Supplemental Information 2 [file peerj-12-18571-s002.pdf]

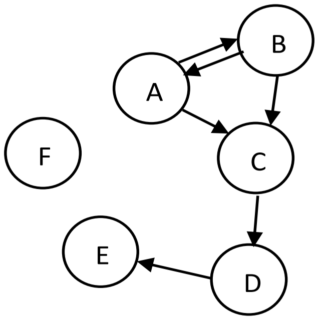

Indegree (A): 1<br>Indegree (B): 1<br>Indegree (C): 2<br>Indegree (D): 1<br>Indegree (E): 1<br>Indegree (F): 0	Outdegree (A): 2<br>Outdegree (B): 2<br>Outdegree (C): 1<br>Outdegree (D): 1<br>Outdegree (E): 0<br>Outdegree (F): 0
Harmonic closeness (A) = (1/1+1/1+0+0+0) = 2<br>Harmonic closeness (B) = (1/1+1/1+0+0+0) = 2<br>Harmonic closeness (C) = (0+0+1/1+0+0) = 1<br>Harmonic closeness (D) = (0+0+0+1/1+0) = 1<br>Harmonic closeness (E) = (0+0+0+0+0) = 0<br>Harmonic closeness (F) = (0+0+0+0+0) = 0	Betweenness (A) = 0<br>Betweenness (B) = 1<br>Betweenness (C) = 2<br>Betweenness (D) = 1<br>Betweenness (E) = 0<br>Betweenness (F) = 0
