# Supplementary material for: Understanding the progress of COVID-19 transmission in a rural district: a social network approach
Source: PeerJ. 2024 Nov 28;12:e18571. doi: 10.7717/peerj.18571 (PMC11608564; doi:10.7717/peerj.18571)
Supplement: Supplemental Information 3 [file peerj-12-18571-s003.pdf]

Supplementary Article S1

Important terminology

Indegree is the total incoming links into a node of a directed network. It refers to the number of confirmed contacts an individual has with a positive patient. Meanwhile, *outdegree* is the opposite of indegree. It is the number of outgoing links from a source (positive case) to another person.[1]

Degree centrality is the number of links of a node. Higher number refers to the node being more connected in the network.

Betweenness centrality is the number of times a node is situated in the shortest path between other nodes. It reflects a patient's role in bridging the transmission of infection between patients who would not have had direct contact with one another.[2]

Closeness centrality is the average shortest path a node has to every other node in the network. It is measured as the inverted sum of the distances from the node to all other nodes and reflects the efficiency of a node in transmitting infection. However, closeness centrality only works in a connected network. In an unconnected network, *harmonic closeness centrality* (HCC) is the better to measure closeness centrality. It is measured as the sum of inverted distances of a node to every other node.[1]

The *clustering coefficient* expresses how many nodes in a graph tend to cluster together. It is also known as *transitivity* and measured as proportion of triples in the network.

Network reciprocity is calculated as fraction of outgoing where there are also ingoing links to the whole links available in the network. It refers to the mutual connection between patients.

Network density refers to the proportion of possible number of connections a node might have. It is calculated as the number of existing ties between nodes, divided by the number of possible ties. Density reflects on the speed of infection might spread provided favourable condition.

Network diameter is the length of the longest shortest path between two connected nodes.

A *clique* is maximum complete subgraph of three or more nodes, which are adjacent to each other.

A network *component* is an isolated grouping of interconnected nodes that are not connected to the rest of the network's nodes. Many networks are made up of a single large component, which is sometimes combined with several smaller ones and a singleton actor.[1]

A *community* is a partition of vertices (cluster) with high edge density.

Super-spreader (operational definition): Any node with an outdegree ⩾5 was considered a super-spreader. It refers to any person who infected five or more people as super-spreader agent.[1]

References

1 Saraswathi S, Mukhopadhyay A, Shah H, Ranganath TS. Social network analysis of COVID-19 transmission in Karnataka, India. *Epidemiol Infect* 2020; **148**: 1–10.

2 Razak FA, Zamzuri ZH. Modelling Heterogeneity and Super Spreaders of the COVID-19 Spread through Malaysian Networks. *Symmetry 2021, Vol 13, Page 1954* 2021; **13**: 1954.
