# Supplementary material for: Understanding the progress of COVID-19 transmission in a rural district: a social network approach
Source: PeerJ. 2024 Nov 28;12:e18571. doi: 10.7717/peerj.18571 (PMC11608564; doi:10.7717/peerj.18571)

**i. Outdegree ≥ 12**

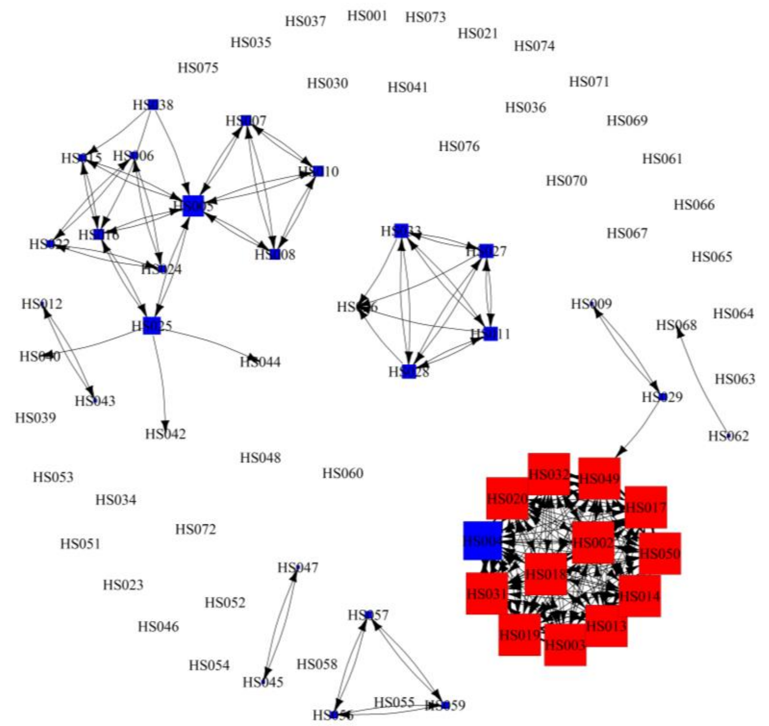


**ii. Super-spreader**

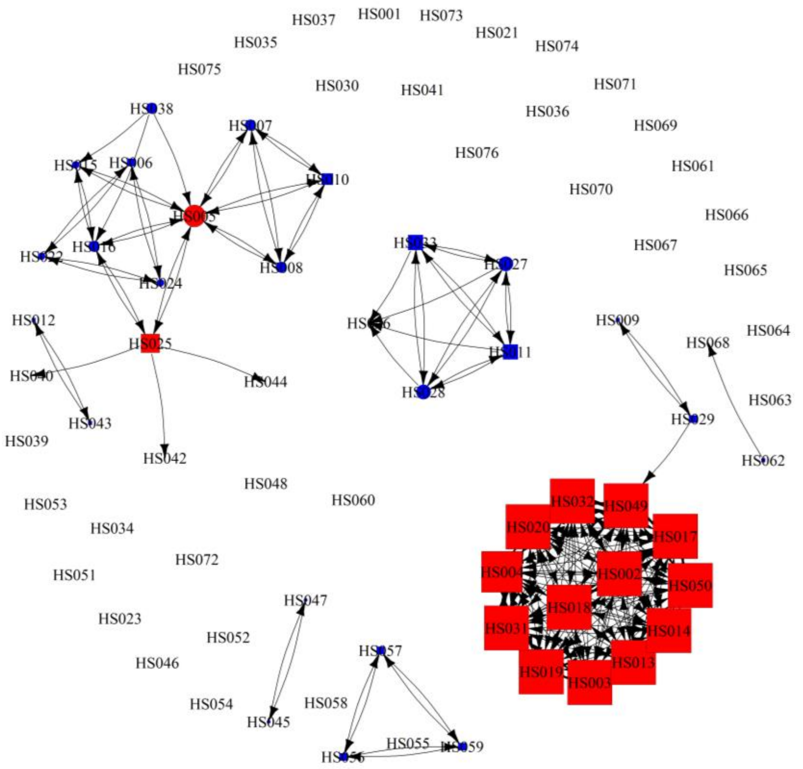

Supplement: Supplemental Information 4 — Figure (i) Nodes in red colour have an outdegree ≥ 12, which constitutes above 95th percentile. Figure (ii) Nodes in red colour are classified as super-spreader. For both figures, square shape denotes male, while circle denotes female. [file peerj-12-18571-s004.pdf]
