# Supplementary material for: Understanding the progress of COVID-19 transmission in a rural district: a social network approach
Source: PeerJ. 2024 Nov 28;12:e18571. doi: 10.7717/peerj.18571 (PMC11608564; doi:10.7717/peerj.18571)
Supplement: Supplemental Information 6 — Table shows the range of degree and betweenness centralities value with their respected average values in the bracket. [file peerj-12-18571-s006.pdf]

Supplementary Table S1

Attributes	Degree	Betweenness
Gender		
- Male	0 – 25 (7.60)	0-24.09 (1.23)
- Female	0 – 13 (2.60)	0-34.5 (1.39)
Ethnicity		
- Malay	0 – 24 (6.31)	0 – 34.5(1.58)
- Chinese	0 (0)	0 (0)
- Indian	0 (0)	0 (0)
- Orang Asli	0 (0)	0 (0)
Age group		
- 0-4 years old	0 (0)	0 (0)
- 5-12 years old	0 – 7 (1.67)	0 – 21 (2.33)
- 13-17 years old	0 – 7 (1.88)	0 (0)
- 18-29 years old	0 – 24 (7.71)	0 – 0.09 (0.02)
- 30-39 years old	0 – 24 (9.07)	0 – 4.50 (0.35)
- 40-49 years old	0 – 5 (1.38)	0 – 13 (1.65)
- 50-59 years old	0 – 24 (5.27)	0 – 34.50 (3.14)
- 60-69 years old	0 – 25 (8.14)	0 – 24.09 (3.45)
- 70-79 years old	24 (24)	0.09 (0.09)

Table S1. Degree and betweenness centralities value. Table shows the range of degree and betweenness centralities value with their respected average values in the bracket.
